# Studying and learning

**DOI:** 10.1111/1753-0407.13379

**Published:** 2023-03-07

**Authors:** Zachary Bloomgarden

**Affiliations:** ^1^ Department of Medicine, Division of Endocrinology Diabetes and Bone DiseaseIcahn School of Medicine at Mount Sinai New York NY USA

A lovely aphorism attributed to Confucius, “学而时习之, 不亦悦乎,” can be translated, “Studying and, from time to time, learning, is it not a pleasure?” This certainly applies to the immense and constantly growing medical literature addressing the many aspects of diabetes. Here are summaries of some recent articles.

## 1 CARDIOVASCULAR DISEASE (CVD) OUTCOMES

The American Diabetes Association “Standards of Care in Diabetes 2023” updated lipid targeting, suggesting that people with diabetes aged 40–75 at higher CV risk should be treated with high‐intensity statins targeting low‐density lipoprotein (LDL)‐cholesterol <70 mg/dL, with the recommendation that ezetimibe or a PCSK9 inhibitor be added to maximum tolerated statins for LDL cholesterol ≥70 mg/dL up to age 75.^1^ Using the American College of Cardiology/American Heart Association atherosclerotic CVD (ASCVD) risk calculator recommended in the document,^2^ the projected 10‐year ASCVD risk of a 50‐year‐old white male with diabetes and treated hypertension with LDL cholesterol 130 is 8.2%, decreasing to 6.2% with statin treatment; it is intriguing to speculate that with the potential 16%–18% further LDL reduction seen with very intensive LDL reduction this might be further reduced to 5.1%. Perhaps 70 is not low enough! A metaanalysis of 10 randomized trials of intensive LDL‐cholesterol lowering lasting at least 3 months compared outcomes in 38 427 persons achieving LDL‐C <40 mg/dL versus 70 668 comparators, finding no significant difference in overall adverse events, adverse events leading to drug discontinuation, cancer, hemorrhagic stroke, new‐onset diabetes, neurocognitive disorders, cataracts, or liver or muscle disorders, either with PCSK‐9 inhibitors, statin/ezetimibe, or cholesteryl ester transfer protein inhibitors, and overall CVD and major adverse cardiovascular event end points were 16% and 18% lower, respectively, in the group reaching this very low level.^3^

What is the optimal glucose‐lowering agent for persons with diabetes and CVD? In a propensity score–matched study of metformin‐treated persons with diabetes in the Taiwanese Diabetes Mellitus Health Database from 2006 to 2017, those taking high sulfonylureas with high (glyburide and glipizide) versus low (gliclazide and glimepiride) affinity to the adenosine triphosphate–sensitive potassium channel had 18% higher risk of major adverse cardiovascular events, 27% higher all‐cause mortality risk, and 82% greater risk of severe hypoglycemia.^4^ An electronic health record analysis of >300 000 persons with type 2 diabetes and CVD found that use of sodium‐glucose cotransporter 2 inhibitors (SGLT2i) and glucagonlike peptide‐1 receptor agonists (GLP‐1RA) increased from ~11% in 2018 to 23% in 2021. Dipeptidyl peptidase 4 inhibitors were used by 15%–18%, and, although the most use was of metformin (~60%) and sulfonylureas (25%–30%), the doubling of use of SGLT2i and GLP‐1RA suggests a real change in practice patterns.^5^

## 2 DIET AND EXERCISE

Among >90 000 persons in the United Kingdom who wore an accelerometer for 7 days at mean age 62, those in the lowest, middle, and highest physical activity tertiles had energy expenditure of 30, 41, and 54 kJ/kg/day (1 kJ is approximately 0.24 kcal). The likelihood of developing type 2 diabetes over the subsequent decade was 3.7%, 1.9%, and 1.1% in the three groups, and each 5jK/kg/day of energy expenditure, the equivalent of a 20 min brisk walk, was associated with a reduction in the likelihood of developing type 2 diabetes by 19% (11% adjusting for body mass index [BMI]).^6^ A 3‐month trial of alternate‐day “fasting,” eating 600 kcal every other day just at dinner, with both the diet and nondiet groups exercising 60 min 5 days weekly, showed that the diet led to lower liver fat, weight, waist, and hepatic transaminase levels and to increased insulin sensitivity.^7^ In follow‐up of nondiabetic obese persons completing a 1‐year moderate or vigorous exercise program, compared with nonexercise controls, glycosylated hemoglobin (HbA1c) decreased from 6.01 to 5.86 and 5.96 at 2 and 10 years, while increasing from 6.03 to 6.25 in the controls, a significant difference. At 10 years the likelihood of developing diabetes was reduced by half: 30 of 74 controls but 29 of the 146 in the intervention group had developed diabetes.^8^

What about developments in pharmacotherapy for obesity? In a 19‐day study of 17 versus 11 persons, all with a 1000‐calorie daily dietary energy deficit (based on calorimetry), receiving placebo versus the glucagon agonist/GLP‐1RA SAR425899, decreased 3.7 versus 4.8 kg, with corresponding 1.8 versus 2.4 kg decreases in fat mass. Although the SAR425899 group had greater weight loss, their reduction in metabolic rate was less than that in the placebo group, along with greater fat oxidation and ketogenesis, suggesting that the usual metabolic adaptation whereby weight loss reduces energy expenditure was blunted by the intervention.^9^ An ongoing concern has been the association of weight loss by obese persons with approximately as much reduction in lean body mass as in fat mass, as was seen in the SAR425899 study. The myostatin/activin type II receptor (ActRII) mediates the inhibition of differentiation of skeletal muscle induced by myostatin or activin A^10^ and may play a role in a variety of states of weight loss and loss of muscle mass. A 48‐week study of individuals with type 2 diabetes either not receiving glucose‐lowering agents or treated with metformin or a dipeptidyl peptidase 4 (DPP4) inhibitor, and having BMI 28–40 kg/m^2^, randomized 58 persons to diet and exercise counseling with monthly infusion of placebo versus bimagrumab, a human monoclonal antibody to ActRII.^11^ Those receiving placebo lost 0.5% fat mass and 0.8% lean mass, whereas with bimagrumab fast mass decreased 20.5%, and lean body mass increased 3.6%, with 0.5 cm increase versus 9.0 cm decrease in waist circumference. A total of 10% receiving placebo versus 65% receiving bimagrumab lost ≥5% of body weight, and HbA1c was unchanged versus decreasing 0.8% from a baseline of 7.8%.

## 3 NEUROLOGIC COMPLICATIONS

Among 57 773 Medicare beneficiaries with new diagnosis of hypertension, 2.2 cases per 100 person‐years of those receiving angiotensin II receptor type 1 blockers, dihydropyridine calcium channel blockers, and thiazide diuretics developed Alzheimer's disease (AD) over a mean of 6.9 years, whereas 3.1 AD cases per 100 person‐years occurred among those receiving angiotensin‐converting enzyme inhibitors, β‐blockers, and nondihydropyridine calcium channel blockers. Vascular dementia was less common but also significantly less frequent in the former group (0.28 vs. 0.46 cases per 100 person‐years, respectively).^12^ The authors ascribe the difference between the groups to the antihypertensive medication regimen exclusively stimulating versus inhibiting type 2 and 4 angiotensin II receptors, and note that there were similar observations in the Prevention of Dementia by Intensive Vascular Care (PreDIVA) trial^13^ and the Systolic Blood Pressure Intervention Trial (SPRINT).^14^ In a review of reviews of efficacy of antidepressants for neuropathic pain, serotonin‐norepinephrine reuptake inhibitors (duloxetine, milnacipran, venlafaxine, and desvenlefaxine) showed moderate certainty evidence and tricyclic antidepressants (amitryptiline, desipramine, imipramine, maprotiline, and nortriptyline) showed low certainty evidence of efficacy, although it is uncertain whether effects measured with a linear pain scale equate to clinical benefit.^15^

## 4 DIABETIC KIDNEY DISEASE

In a metaanalysis of 17 studies of effect on albuminuria of an SGLT2i, a mineralocorticoid antagonist (MRA), both, or neither, including 34 412 patients with urine albumin/creatinine ratio (UACR) >30 mL/min/1.73 m^2^, combination treatment reduced UACR 65%, whereas SGLT2i and MRA alone reduced UACR 34% and 32%, respectively. Compared with placebo, SGLT2i, MRA, and the combination reduced systolic blood pressure 3, 3, and 9 mm Hg, respectively.^16^ A somewhat different conclusion was reached in a randomized crossover trial of 63 persons with diabetes (26 type 1 and 37 type 2) and microalbuminuria (mean UACR 115) treated over sequential 4 week periods with 4‐week washouts. Reduction in UACR >30% was seen in 49% of those receiving telmisartan 80 mg (particularly among those receiving a diuretic at baseline), in 25% of those receiving empagliflozin 10 mg daily, in 21% of those receiving linagliptin 5 mg daily, and in 10% of those receiving the JAK–STAT inhibitor baricitinib 2 mg daily; among those not showing such a reduction in UACR with telmisartan, 28%, 21%, and 9% showed a >30% reduction in UACR with empagliflozin, linagliptin, and baricitinib, respectively, and 27% did not show such a reduction in UACR with any of the agents.^17^ The study reminds us not to lose sight of the benefit of angiotensin‐directed agents in the current excitement about the SGLT2i and MRA classes, although the particular benefit in persons receiving diuretics and the use of the lower empagliflozin dose may have somewhat skewed the findings. A further implication is that the degree of reduction in albuminuria varies from person to person with different agents, suggesting that in clinical practice one might carefully try a variety of agents to optimize each patient's response. In a 52‐week randomized controlled trial of continuous positive airway pressure (CPAP) treatment of 185 persons with obstructive sleep apnea (apnea‐hypopnea index [AHI]) ≥10/h and diabetic kidney disease (UACR >30 mg/g, 24‐h urine albumin ≥30 mg, or estimated glomerular filtration rate < 60 mL/min/1.73 m^2^), although there was no significant improvement in the primary UACR end point in an intention to treat analysis, only 47 of the 93 persons randomized to CPAP used it ≥4 h/night. Those who did have good adherence to CPAP treatment had a modest 10% decrease in UACR, particularly among those with AHI >30/h. HbA1c decreased 0.4% and, among participants not treated with insulin, homeostatic model assessment of insulin resistance improved as well,^18^ further supporting potential benefit of this treatment in appropriate patients.

## 5 HYPOGLYCEMIA

In the 6.3‐year CARdiovascular Outcome study of LINAgliptin versus glimepiride in patients with type 2 diabetes (CAROLINA), 3014 persons received linagliptin 5 mg daily versus 3000 glimepiride 1–4 mg daily; 89% also were treated with metformin. Glimepiride and linagliptin were associated with similar mean HbA1c patterns over the course of the study, but glimepiride led to 1.5 kg greater mean weight over the course of the study and to a 4.7 to 6.3‐fold greater likelihood of total hypoglycemia events (depending on the definition used). Symptomatic or asymptomatic hypoglycemia with glucose <54 mg/dL or severe hypoglycemia (requiring assistance of another person) occurred during the night in 63 persons receiving linagliptin versus 145 receiving glimepiride, and such episodes during the day were reported in 133 versus 1164, respectively.^19^ In a study of insulin clamp‐induced hypoglycemia (mean 45, nadir 38 mg/dL) in 24 persons with type 1 diabetes, epinephrine and norepinephrine increased, heart rate increased, potassium decreased during hypoglycemia and increased above baseline during recovery, and the QTc interval on electrocardiogram increased, in 15 of the participants exceeding 500 msec, and remained prolonged during recovery either to eu‐ or to hyperglycemia, suggesting arrhythmia risk during and following the hypoglycemia period.^20^ Given these concerns, novel approaches to preventing hypoglycemia are welcome. In a study of persons with type 1 diabetes using continuous glucose monitoring over 795 exercise sessions lasting 30–75 min, administration of 150 mcg of a liquid‐stable glucagon preparation before exercise was associated with 12% occurrence of glucose ≤70 during or within 30 min following exercise whereas 39% of persons receiving a placebo injection had hypoglycemia, with both groups reducing their insulin pump infusion rate by 50% reduction during the exercise period; another group receiving glucagon without reducing insulin had a comparable 16% hypoglycemia rate during or immediately following exercise.^21^
